# Mesenchymal Stromal Cells for the Treatment of Interstitial Lung Disease in Children: A Look from Pediatric and Pediatric Surgeon Viewpoints

**DOI:** 10.3390/cells10123270

**Published:** 2021-11-23

**Authors:** Gloria Pelizzo, Serena Silvestro, Maria Antonietta Avanzini, Gianvincenzo Zuccotti, Emanuela Mazzon, Valeria Calcaterra

**Affiliations:** 1 Pediatric Surgery Department, Children’s Hospital “Vittore Buzzi”, 20154 Milano, Italy; 2 Department of Biomedical and Clinical Sciences-L. Sacco, University of Milan, 20157 Milan, Italy; gianvincenzo.zuccotti@unimi.it; 3 IRCCS Centro Neurolesi “Bonino-Pulejo”, Via Provinciale Palermo, Contrada Casazza, 98124 Messina, Italy; serena.silvestro@irccsme.it (S.S.); emanuela.mazzon@irccsme.it (E.M.); 4 Cell Factory, Pediatric Hematology Oncology Unit, Fondazione IRCCS Policlinico San Matteo, 27100 Pavia, Italy; ma.avanzini@smatteo.pv.it; 5 Department of Pediatrics, Children’s Hospital “Vittore Buzzi”, 20154 Milano, Italy; valeria.calcaterra@unipv.it; 6 Pediatrics and Adolescentology Unit, Department of Internal Medicine, University of Pavia, 27100 Pavia, Italy

**Keywords:** mesenchymal stromal cells, children, interstitial lung disease, pediatrics

## Abstract

Mesenchymal stromal cells (MSCs) have been proposed as a potential therapy to treat congenital and acquired lung diseases. Due to their tissue-regenerative, anti-fibrotic, and immunomodulatory properties, MSCs combined with other therapy or alone could be considered as a new approach for repair and regeneration of the lung during disease progression and/or after post- surgical injury. Children interstitial lung disease (chILD) represent highly heterogeneous rare respiratory diseases, with a wild range of age of onset and disease expression. The chILD is characterized by inflammatory and fibrotic changes of the pulmonary parenchyma, leading to gas exchange impairment and chronic respiratory failure associated with high morbidity and mortality. The therapeutic strategy is mainly based on the use of corticosteroids, hydroxychloroquine, azithromycin, and supportive care; however, the efficacy is variable, and their long-term use is associated with severe toxicity. The role of MSCs as treatment has been proposed in clinical and pre-clinical studies. In this narrative review, we report on the currently available on MSCs treatment as therapeutical strategy in chILD. The progress into the therapy of respiratory disease in children is mandatory to ameliorate the prognosis and to prevent the progression in adult age. Cell therapy may be a future therapy from both a pediatric and pediatric surgeon’s point of view.

## 1. Introduction

Stem cell therapy represents a prospective approach in regenerative medicine for the repair, replacement, and rejuvenation of tissue [1,2]. Mesenchymal stromal cells (MSCs) expanded in vitro have been proposed as potential therapy to treat congenital and acquired lung diseases [2,3,4,5,6,7,8,9,10,11]. MSCs are multipotent cells that can differentiate into multiple tissue-forming cell lineages, such as osteoblasts, adipocytes, chondrocytes, tenocytes, and myocytes. In addition, MSCs regulate immune and inflammatory responses. MSCs are characterized by an innate self-renewal capacity, and they can be in vitro expanded without losing their differentiation potential [12].

The lung originates from the endodermal and mesodermal germline. Each phase in lung development is reliant on inductive cues and reciprocal interactions between the pulmonary epithelium and the surrounding mesenchyme. Loss of or abnormalities in cells and in their interactions can lead to severe anatomical and functional defects in the airway and alveoli [13,14,15,16]. MSCs are key cells in the connective pulmonary tissue hierarchy, supporting the crucial relationship between the epithelium and mesenchyme during branching morphogenesis [13,14,15,16].

Interstitial lung disease in children (chILD) is a group of highly heterogeneous and rare respiratory diseases with wide ranges in the age of onset and disease expression [2,17,18,19]. chILD is characterized by inflammatory and fibrotic changes in the pulmonary parenchyma, leading to gas exchange impairment and chronic respiratory failure associated with high morbidity and mortality. chILD disorders often overlap, and the classifications remain difficult. chILD is characterized by abnormality of the lung interstitium, alveoli, and distal air spaces, leading to chronic respiratory failure. Even though chILD pathogenesis remains unclear, the aberrant activation of the alveolar epithelium and mesenchyme has been proposed as a crucial player [18,20]. A multidisciplinary approach to the diagnosis and follow-up is mandatory to provide improved care. Surgery may represent a critical step for management. The therapeutic strategy is mainly based on the use of corticosteroids, hydroxychloroquine, azithromycin, and supportive care; however, the efficacy is of these methods is variable. The role of MSCs as a state-of-the-art treatment has been proposed in clinical and preclinical studies [4,21,22,23,24,25,26,27,28,29].

This narrative review reports the information currently available on MSCs treatment as a new therapeutic strategy in chILD. To evaluate the efficacy of MSCs treatment, the pre-clinical and clinical results on bronchopulmonary dysplasia (BDP) were also included as model of chronic respiratory diseases characterized by interstitial lung abnormalities leading to chronic failure. The need for research to advance the therapy of pulmonary diseases is mandatory to improve the prognosis and to prevent their progression in adulthood. Cell therapy, as reparative and regenerative treatment, may be innovative from both the pediatric and pediatric surgeon’s viewpoint.

## 2. Methods

A narrative review of the English literature published in the past 10 years was conducted. We independently identified the most relevant published manuscripts including original papers, metanalyses, clinical trials, and reviews. Case reports or series and letters were excluded. Human and experimental studies were included. Papers were searched using the following keywords in combination: “children interstitial lung disease and mesenchymal stromal cells”, “children idiopathic pulmonary fibrosis and mesenchymal stromal cells” and “infants bronchopulmonary dysplasia and mesenchymal stromal cells and clinical trials”. The following electronic databases were searched: PubMed, Scopus, EMBASE, and Web of Science. In this study, we included clinical studies recorded on ClinicalTrial.gov searched with the following keywords in combination: “stem cells” and “infants bronchopulmonary dysplasia”. The contributions were critically reviewed and collected. The final version was approved by all authors.

## 3. Mesenchymal Stromal Cells and MSCs-Derived Extracellular Vesicles

Cell therapy has recently received considerable interest as a treatment of respiratory system diseases. Several lines of evidence encourage the use of MSCs in lung diseases [30,31]. The ease of isolation, the lack of immunogenicity, and the ability to expand ex vivo and to differentiate in multilineages make MSCs an attractive therapeutic tool. The therapeutic potential of MSCs is due to their paracrine effect, supported by their secretion of extracellular vesicles (EVs), transferring genetic material, and releasing of soluble factors such as cytokines. The conditioned medium (CM) or secretome is defined as the products secreted by MSCs in their cultured medium [32,33,34]. In Vivo administration of MSCs, as well as of their products, appears a suitable approach for injured lung tissue repair by reducing fibrosis and stimulating proper alveolar and vascular repair [35,36].

### 3.1. Mesenchymal Stromal Cells (MSCs)

MSCs are a population of multipotent nonhematopoietic stromal cells, capable of differentiating into tissue derived from the three main mesodermal lineages. MSCs represent a cell population with secretion [37], homing [38], and immunomodulatory [39] properties.

MSCs can be isolated from various sources, including bone marrow, adipose tissue, skeletal muscle, synovium, spleen, thymus, lung, and amniotic fluid. The International Society for Cell and Gene Therapy reported, as minimal criteria for their definition: plastic adherence when in vitro cultured; the expression of surface markers such as CD105, CD73, and CD90; and the absence of the expression of endothelial and hematopoietic markers such as CD11b, CD14, CD19, CD34, CD45, CD79a, and class II human leukocyte antigen-DR (HLA-DR) [40,41]. They have to show the capability to differentiate in vitro into tissues of mesodermal origin, such as osteoblasts, adipocytes, and chondroblasts. Subsequently, it was shown that MSCs can transdifferentiate into neural cells, pancreatic cells, liver cells, and cardiomyocytes [42].

MSCs can exert several functions including immunomodulatory effects through inhibition of T-cell proliferation and secretion of anti-inflammatory cytokines and growth factors [43,44]. In Vivo, their effects were reported to be mediated by paracrine mechanism with the release of growth factors, which stimulate endogenous repair pathways [12] and host inflammation sites that express anti-inflammatory cytokines to dampen the host’s immune response [45].

It is a long-established fact that MSCs infuse intravenously; while circulating within the lungs, they remain partially entrapped, producing the pulmonary first-pass effect. This effect represents one of the hurdles faced by MSCs therapy when targeting other organs, but may be an inherent advantage when biotherapy with MSCs is directed to the lungs. The ability to deliver MSCs to the lungs via a simple intravenous approach has the potential for large-scale retention [11,46,47]. Even more attractively, retained cells appear to target areas of injured lung where they differentiate into specific cell types and start regeneration [48]. Both in vitro and in vivo, MSCs have been observed to differentiate into alveolar epithelial cells, indicating their potential as a regenerative therapy for lung diseases [49,50,51].

Isolation and culture make MSCs suitable candidates for preclinical and clinical studies. Effectively, clinical trials phase I in patients with chronic obstructive pulmonary disease (COPD) confirmed the safety of MSCs; outcomes from phase I/II clinical trial administration and investigation showed their potential anti-inflammatory effects, reporting a reduction in C-reactive protein levels [52].

### 3.2. Extracellular Vesicles (EVs)

MSCs also exert their therapeutic effects through the release of EVs. MSCs-derived EVs are small membrane-bound vesicles that contain biomolecules including proteins, lipids, microRNA, and mRNA, which play a role as mediators in cellular communication to maintain physiological homeostasis [53].

EVs provide intercellular communication by transferring their membrane contents to the target cells through the binding of surface receptors [54,55]. EVs, according to their origin, are divided into microvesicles and exosomes [56]. Microvesicles are generated by direct budding of the plasma membrane toward the extracellular environment through a calcium-induced asymmetric reorganization of phospholipids [57]. Membrane germination is induced by calpain and gelsolin, proteases that cut the protein network of the cytoskeleton [58]. The exosomes instead originate from the intracellular fusion of the endosomes with the endocytic vesicles, thus incorporating their contents. After maturation, endosomes merge with the plasma membrane, becoming exosomes that are released into the extracellular space [59]. The microvesicles are larger, ranging from 50 to 1000 nm; exosomes are smaller, with a size ranging from 40 to 120 nm [60,61]. Western blot and mass spectrometry analyses allowed the identification of proteins expressed in EVs [60,61]. In particular, microvesicles contain phosphatidylserine, metalloproteinases, some integrins, and P-selectin [62]. Exosomes contain proteins with GTPase activity involved in transport and fusion [63], heat shock proteins (Hsp 60, Hsp70, Hsp90) [59,63], and tetraspanins (CD63, CD81, and CD9), which are necessary in the fusion between exosomes and recipient cells [64]. EVs’ composition and structure are different according to the mother cell. Moreover, EVs mediate horizontal mRNA transfer to a recipient cell [59,65]. As such, EVs are able to change the phenotype and function of the recipient cells, regulating different cellular pathways and activating regenerative mechanisms. Therefore, MSC-derived EVs could be capable of restoring the homeostasis of damaged tissues [66] and interacting with immune system cells [67], demonstrating regenerative and anti-inflammatory properties [68]. The use of EVs confers some advantages compared to the use of original stem cells, such as a higher safety profile, an increased ability to cross biological barriers, lower immunogenicity, and poor immune rejection. Despite the advantages shown by MSC-derived EVs, their use in clinical studies requires further investigation to resolve critical issues related to the methods of production, characterization, quantification, pharmacokinetics, and transfer to target sites [69].

### 3.3. Conditioned Medium (CM)

The CM/secretome from cultured MSCs, containing both soluble factors and EVs, may represent a significant tool to produce efficacy similar to the original cells [70]. A systematic review and meta-analysis of preclinical studies, including different lung diseases such as bronchopulmonary dysplasia, asthma, pulmonary hypertension, acute respiratory distress syndrome, chronic obstructive pulmonary disease, and pulmonary fibrosis, recorded comparable efficacy between CM and MSCs [71]. To obtain CM from cultured MSCs, different collection timings, confluence grades, and culture passages have been reported in the literature [72,73,74]. As already underlined, MSC-derived CM consists of all MSC-secreted cytokines, proteins, growth factors, and EVs [75]; it contains several angiogenic growth factors, such as vascular endothelial growth factor (VEGF) [76,77]. In in vitro models, it was shown that MSC-derived CM containing high levels of VEGF promoted angiogenesis and the regeneration of periodontal tissue [78]. In another study, the addition of anti-VEGF antibodies to MSC-derived CM decreased vessel formation and induced poor bone regeneration in a rat calvaria model [77].

In line with these findings, in a mouse model of *Escherichia coli* endotoxin-induced model of acute lung injury (ALI), administration of MSC-derived CM induced a reduction in septal thickening, alveolar hemorrhage, alveolar infiltrates, and fibrin filaments compared to untreated mice. Similar reductions in neutrophils and lung permeability were observed both after treatment with MSCs or CM [79]. Moreover, in a rat model of bleomycin-induced pulmonary fibrosis, administration of MSC-derived CM reduced the deposition of collagen involved in fibrosis [80].

Therefore, these data encourage the use of the CM as potential cell-free therapy; however, it is necessary to identify and standardize the most advantageous cellular source, the culture method, and the purification method to obtain the maximum therapeutic yield.

## 4. Routes of Administration of MSCs

The use of MSCs as treatment for respiratory diseases, although promising, needs to overcome some limitations. Some of these include determining the optimal dosage, the cell type, and the appropriate route of administration.

In preclinical and clinical studies, cell therapy envisages systemic administration, such as intravenous or intra-arterial infusion or local administration via intratracheal administration [81]. Intravenous cell infusion causes the accumulation of MSCs in the lungs, an effect known as the first pass [82]. This phenomenon may represent an obstacle when MSCs therapy is aimed toward other organs but may be a notable advantage when MSC therapy is directed to the lungs. However, it has been shown that this entrapment is transient, followed by a distribution of the cells to other organs, such as the spleen and liver, during the following 24–48 h [83,84,85].

In an in vivo study performed in a rodent silicosis model with intravenously administered bone-marrow-derived MSCs (BM-MSCs), the first pass event was observed six hours after treatment [86], whereas in a rodent model of myocardial infarction, it occurred within minutes with subsequent embolization [87]. However, the small diameter of the pulmonary microcirculation and the presence of adhesion receptors may prevent MSCs from reaching the targeted lung site [88]. MSCs entrapment in the vascular system can induce the formation of an embolus, as demonstrated by a clinical study in which cell therapy promoted venous thrombosis in two patients with Crohn’s disease [89]. Instead, intra-arterial infusion allows reaching the target organ, avoiding entrapment inside the lung [81,82,90]. However, even this procedure may be harmful due to the possible generation of emboli in microcirculation [91].

The local administration has greater or equal therapeutic potential at lower dosages than systemic administration [92]. For cell therapy, local delivery to the lungs occurs by intratracheal administration [81]. This route, although a possible cause of trauma and injury, has been reported to produce promising therapeutic outcomes in several clinical studies [36,93,94], showing MSCs engraftment into the lung in real time [88]. However, the available data are contradictory and insufficient to identify the most suitable route for MSC infusions both in terms of safety and of targeted delivery to a specific area of the organ, in particular in the lung.

## 5. Interstitial Lung Disease in Children (chILD)

chILD refers to a heterogeneous group of rare lung disorders, with a wide range of prevalence (from 0.13 to 16.2/100,000 children/year) as a result of a lack of standardized diagnostic criteria, and a heterogeneous clinical presentation and pathological picture [17,18]. chILD is characterized by abnormalities in the lung interstitium, alveoli, and distal air spaces, leading to abnormal gas exchange and chronic failure [17,18].

The pathogenesis of chILD is complex and has not yet been fully elucidated. The central role of the alveolar epithelium and aberrant mesenchymal activation has been proposed [18,20]. The involvement of repeated injuries of vulnerable alveolar epithelial cells (AECs) and the failure of the alveoli to respond to injury, leading to aberrant lung repair and progressive fibrosis, were suggested to be involved in pathogenesis [95]. An acceleration of the ageing process of progenitor cells, leading to stem cell exhaustion, was also proposed [96]. The initial recruitment of inflammatory cells including collagen-producing fibrocytes is not excluded in the pathophysiology of alveolar injury.

chILD comprises more than 200 different conditions, for which different classification systems have been proposed based on the etiology and physiopathology and lung biopsies [18,20]. As reported in Table 1, more recently, a subclassification considering infancy ILD different from other pediatric ILD was introduced, again on the basis of etiologic and pathologic criteria [17,18,97,98,99].

In most of the chILD cases, no family history is documented; moreover, the occurrence of familial forms with an estimated prevalence ranging from 1.3 to 5.9 per million has been reported [17,18]. Mutations in the surfactant protein (SP) genes, mainly in SP-B and -C genes, are responsible for the familial form [17,18]. As reported in Table 2, other genetic forms have been described.

The clinical presentation of chILD varies, ranging from mild nonspecific symptoms to a very severe clinical picture. Usually, the earlier the disease onset, the more severe the presenting symptoms [100].

During the neonatal period, in term neonates, chILD may occur shortly after birth, with unexplained respiratory distress requiring intubation and ventilation [101]. In born preterm infants, chILD presents with acute respiratory distress that is more severe and is expected because of prematurity [101].

During the first two years of life [102,103], common presentations include asymptomatic forms or nonspecific respiratory signs and symptoms, such as dyspnea, polypnea, dry cough, wheezing, recurrent respiratory infections, and exercise intolerance [101]. Moreover, severe respiratory distress usually triggered by viral infections may occur. Older children can show tachypnoea, hyperoxia, digital clubbing, and/or cyanosis during exercise or at rest [101,104,105]. As reported in Table 3, a severity score of disease was proposed by Fan et al. [106].

At the initial investigation, the chest radiograph may be normal or reveal nonspecific alterations [107]. The role of functional lung testing in infants is unclear: in older children, a restrictive pattern is usually detected. Within the diagnostic workup, blood tests including genetic evaluation, immunological profile, and autoantibody studies are recommended, and environmental organic dust exposures could be considered.

When chILD is suspected clinically, chest computed tomography (CT) scanning is the gold standard to evaluate the presence and extent of lung damage; however, only in some cases can it be diagnostic [107]. Common radiologic patterns in chILD are widespread ground-glass attenuation, sometimes coupled with intralobular lines; irregular interlobular septal thickening; honeycombing; and, less frequently, large subpleural air cysts (usually located in upper lobes adjacent to areas of ground-glass opacities) [18,108,109,110]. Invasive testing, such as bronchoscopy with bronchoalveolar lavage (BAL), may be diagnostic in pulmonary hemorrhage syndromes, alveolar proteinosis, and eosinophilic lung disease, and a normal cell differential can rule out hypersensitivity pneumonitis [17]. Endobronchial and transbronchial biopsies are rarely performed in chILD and are not recommended unless a specific diagnosis is suspected, such as pulmonary alveolar microlithiasis or sarcoid granulomas. Lung biopsy is the last step in the diagnostic workup. The timing and need for lung biopsy in chILD remain controversial; biopsy prior to steroid treatment is recommended to minimize risk to wound healing and to expedite specific chILD treatments.

A multidisciplinary approach for diagnosis and follow-up is mandatory in chILD. Pediatricians, pediatric pulmonologists, radiologists, geneticists, and pediatric surgeons are the crucial players [17,18].

The prognosis of chILD is variable, ranging from complete recovery in neuroendocrine cell hyperplasia during infancy and pulmonary interstitial glycogenosis to a mortality rate that approaches 100% in alveolar capillary dysplasia. The overall mortality rate is around 15%, with a variable outcome in infants [106,111,112,113,114].

The standard treatment of chILD is mainly supportive and based on oxygen supplementation, and/or ventilation, and respiratory physiotherapy [17,18]. Nutritional support is mandatory to maintain an adequate caloric intake to prevent failure to thrive. From a pediatric surgery point of view, most of these patients require tracheostomy in the few first months of life, followed by enteral nutritional supply by gastrotomy. With time and in order to protect the lung tissue, most of the children need an antireflux procedure. In some patients, when bullous emphysema and hypertensive pneumothorax occur, leading to ventilation failure, surgical resection may be necessary to ensure lung growth.

Empirical medical therapy with anti-inflammatory and immunomodulatory drugs including corticosteroids, hydroxychloroquine, and azithromycin is usually used with varying degrees of efficacy [17,18]. New antifibrotic drugs currently proposed in adults, including pirfenidone and nintedanib, may represent new therapy options for certain forms of chILD in the future. Lung transplant is a therapeutic option for children with end-stage chronic respiratory failure. The outcome and survival are similar to those reported in other lung conditions [115].

Preclinical and clinical studies support a potential beneficial role of cell therapy to prevent the chILD progression [116]. Additionally, cell therapy could be useful to promote the repair and regeneration of the impaired lung.

## 6. Preclinical and Clinical Studies

### 6.1. Preclinical

ILD is a group of diseases that includes several chronic lung diseases characterized by varying degrees of inflammation and fibrosis. Most ILDs are idiopathic, including idiopathic pulmonary fibrosis (IPF). In order to understand the pathogenetic mechanisms of pulmonary fibrosis and identify possible therapeutic targets, several animal models have been developed that are capable of mimicking the human characteristics of the disease. The bleomycin model is the most used and best characterized model that reproduces different cellular and molecular mechanisms involved in IPF and in other fibrotic ILDs [117,118]. Bleomycin is a complex glycopeptide [119] isolated from *Streptomyces verticillus*, an actinobacteria strain [120]. This drug showed usefulness as an anticancer in several carcinomas and lymphomas [121]. However, the use of this drug has been limited due to its toxicity in organs such as the lung. These adverse events promoted the use of this agent to induce animal models of pulmonary fibrosis [122]. Initially, drug administration induces direct damage of alveolar epithelial cells, promoting the production of alveolar inflammatory cells within the first seven days [123]. Subsequently, these cells are eliminated and the proliferation of fibroblasts is induced, with the consequent development of pulmonary fibrosis [124]. Notably, the bleomycin model is characterized by an overlap between the inflammatory and fibrotic models. Initially, the model induces an early inflammatory phase that switches to the fibrotic state after 5–7 days. The strong inflammatory response induced by bleomycin may take up to 10 days before complete elimination. The fibrotic phase begins when the inflammatory process has subsided. After two weeks, the resolution of the fibrosis starts. Therefore, in this animal model, the “time window” during which it is possible to test the fibrogenic mechanisms and the antifibrotic drugs action’s is relatively short [125,126]. Another experimental model that is used is induced by fluorescein isothiocyanate (FITC), which involves the intratracheal administration of FITC in both C57Bl/6 and Balb/c mice [127,128]. FITC caused infiltration of mononuclear and neutrophil cells into the lung interstitium, inducing pulmonary fibrosis by day 21, as also observed in the bleomycin model [128]. Silica-induced lung fibrosis is another model used to reproduce fibrotic nodules that resemble lesions that develop following occupational exposure to mineral dust and particulate aerosols [129]. Silica is trapped in the lung where a toxic and inflammatory response is activated, inducing the alveolal accumulation of proteins and neutrophilia, responsible for activating a fibrotic response [130,131]. Several transgenic strains mimic the characteristics of pulmonary fibrosis, such as human collagenase directed by the haptoglobin promoter [132], PDGF-B directed by the surfactant protein C (SP-C) promoter, human transforming growth factor-alpha (TGF-α) directed by the SP-C promoter [133], interleukin (IL)-11, and IL-13 directed by the bronchiolar exocrine/club cells 10-kDa (CC10) protein promoter [134,135]. The transgenic model, however, has the disadvantage of not accurately recreating the multigenic environment of natural pulmonary fibrosis. Additionally widely used are models involving the use of adenoviral vectors, which exploit gene transfer mediated by adenoviruses to overexpress cytokines and chemokines such as granulocyte-macrophage colony-stimulating factor (GM-CSF) [135], tumor necrosis factor-alpha (TNF-α) [136], transforming growth factor-beta 1 (TGF-β1) [137], and IL-1β [138]. These transgenes persist for 21 days and cause major fibrotic lesions in rodents, proving useful models for studying the pathogenesis of this disease [139]. Contrarily, the use of lentiviral vectors permanently transfers the transgenes in different cells of the lung, thus allowing analysis of the effect of the overexpression of the transgene in the long term. These models are useful for studying the effect of the gene product at the onset and during pulmonary fibrosis [140].

#### 6.1.1. Animal Models of Pulmonary Fibrosis

The use of MSCs in clinical practice is now a field of considerable interest. However, most preclinical studies employed human BM-MSCs to treat asthma [141], acute lung injury [142], pulmonary fibrosis [143], and acute respiratory distress syndrome [144]. In this context, Balogh et al. [145] evaluated the immunomodulatory role of MSCs taken from bronchoalveolar lavage fluid (BALF) from patients with hypersensitivity pneumonitis (HP) compared to normal MSCs isolated from healthy subjects. Immunophenotyping by flow cytometry and confocal laser scanning microscopy demonstrated that BALF MSCs have reduced levels of CD105, CD73, and CD90. Therefore, these cells, compared to the MSCs isolated from control subjects, showed a loss of immunosuppressive activity. In order to evaluate the role of bronchoalveolar MSCs on T-cell function in vitro, phytohemagglutinin (PHA)-stimulated peripheral blood mononuclear cells (PBMCs) were co-cultured with normal or HP-derived BALF MSCs. Measurement of 5,6-carboxyfluorescein-diacetat succinimidyl ester (CFSE) positivity by flow cytometry demonstrated that normal MSCs were able to reduce T-cell proliferation, whereas HP-derived MSCs did not have a significant immunosuppressive effect on normal or HP T lymphocytes. When T cells were cultured with normal MSCs or HP, reduced proliferation and activation of CD4^+^ and CD8^+^ T cells were observed with reduced CD25 positivity mediated by normal MSCs. In contrast, HP-derived MSCs did not induce division energy in either T-cell subtype. Therefore, these results demonstrate that healthy MSCs can affect HP-derived activated T cells, suggesting the use of these cells as a potential therapeutic approach for this type of ILD [145].

Given the positive role of MSC transplantation in reducing pulmonary fibrosis [146], Chen et al. [147] evaluated the effects of adipose-derived MSCs (AD-MSCs) in rats in which pulmonary fibrosis was silica-induced. For the study, AD-MSCs were taken from the adipose tissue of rats and cultured in vitro. In order to study the antifibrotic effects of AD-MSCs, the animals underwent oral-tracheal intubation with a silica suspension (50 mg/mL) to induce the pulmonary fibrosis pattern. After 24 h, the animals were treated intravenously with AD-MSCs (1 × 10^6^ cells/kg). Twenty-eight days after the transplant, the animals were sacrificed and the organs were isolated for histopathological investigations. In lung tissue, treatment with AD-MSCs markedly reduced the expression levels of TNF-α, IL-6, and IL-10; conversely, it increased IL-1β expression levels. Treatment with AD-MSCs reorganized the alveolar structure that had been severely destroyed by exposure to silica, reducing inflammatory cell infiltration phagocytic cells and silicon nodules. Additionally, AD-MSCs reduced silica-induced apoptosis in lung tissue cells. A decrease in caspase-3 was observed due to the decrease in the B-cell lymphoma protein-2-associated X (Bax)/B-cell lymphoma protein 2 (Bcl-2) ratio in the treatment group. These results further confirmed that AD-MSCs appear to exert a lung protective effect and reduce the apoptosis process in the animal model of pulmonary fibrosis [147].

Most preclinical and clinical studies evaluated the efficacy of BM-MSCs and AD-MSCs in IPF. Cores et al. [148] instead investigated the effects of adult lung spheroid cells (LSCs) as a source of stem cells for allogeneic cell therapy in pulmonary fibrosis. For the study, lung outgrowth cells from the distal region of the lungs were harvested from male Wistar-Kyoto rats (MHC haplotype, RT^I^), used as LSCs donors. In order to evaluate the proangiogenic effects in vitro, human umbilical vein endothelial cells (HUVECs) were cultured in media conditioned with LSCs. The data showed that LSCs, by means of paracrine action, stimulated angiogenesis by reducing fibrosis. To reproduce an allogeneic cell transplantation model, Wistar-Kyoto and Brown Norway female rats (MHC haplotype, RT^II^) as syngeneic and allogeneic recipients, respectively, were used for the experiment. The animals were treated with bleomycin (1.5 U/kg) intratracheally on day 0 to induce the pulmonary fibrosis pattern. The following day, the rats were treated with LSCs (5 × 10^6^ cells) intravenously via the tail vein, while the control group received saline. Both allogeneic and syngeneic LSCs alike attenuated the onset of fibrosis and the deposition of connective tissue and collagen in the post-injury lungs. LSC treatment also protected the pneumocytes from bleomycin-induced injury, reduced apoptosis in the lungs, and induced angiogenesis. Expression data on cytokine levels, such as inflammatory cytokine, immune response, wound healing, and epithelial proliferation, demonstrated that the treatment creates no systemic immune or inflammatory response. Furthermore, data on the expression levels of T lymphocytes in the lung showed that cell therapy did not induce any local immune response. Therefore, this evidence suggests that allogeneic LSC treatment can be considered safe and efficacious in slowing the progression and severity of fibrosis [148].

In the above-described studies, MSCs were administered intratracheally or intravenously. MSCs administration appears to decrease inflammation and fibrosis in several chronic lung disease models of newborn rodents. Despite the benefits of this therapeutic approach, the route of administration and the optimal dose in newborns still need to be determined. Liu et al. [149] surveyed the dose-dependent effects of intranasal MSCs release versus the intraperitoneal route in an early neonatal lung injury model. The long-term mechanical and histological effects of human umbilical cord derived MSCs (hUC-MSCs) were evaluated in the study. In order to induce lung damage, neonatal mice with severe combined immunodeficiency were exposed to hyperoxic conditions from birth until postnatal day 7, while one group was maintained in normoxic conditions as a control group. On the fifth postnatal day, during the phase of intense acute lung injury, the pups were subjected to the administration of cell suspension (0.1, 0.5, or 1 × 10^6^ cells/kg in 20 μL of phosphate-buffered saline) via the intranasal or intraperitoneal route. Instead, sham normoxic controls received 20 μL of phosphate-buffered saline either intranasally or intraperitoneally. Animals were sacrificed either 48 h or 8 weeks after cell transplantation to assess the short- and long-term effects of cell therapy. Systemic (intraperitoneal) administration of hUC-MSCs in neonatal mice exposed to hyperoxia restored lung compliance, pressure-volume loop, and elastance in a dose-dependent manner 8 weeks after transplantation. At the highest dose (1 × 10^6^ cells/kg), intraperitoneal MSC transplantation effectively increased the thickness of the alveolar septum, probably remodeling the interstitial matrix. Conversely, transplantation of hUC-MSCs via the intranasal or intraperitoneal route at lower doses had no significant effects on lung function or alveolar remodeling. Therefore, the results of this study highlight the beneficial effects of hUC-MSCs transplantation on tissue recovery and lung function following chronic neonatal injury. However, future studies are needed to evaluate the long-term safety of systemic transplantation with MSCs, especially when considered for pediatric use [149].

Mansouri et al. [150] investigated the therapeutic effects of EVs isolated from BM-MSCs in an animal model of IPF. In order to induce the IPF model, C57BL/6 mice were subjected to a single intratracheal administration of bleomycin sulfate (50 μL, 3 U/kg), while the control group received saline (50 μL). Simultaneously, the animals were treated with a single dose of EVs (200 μL; dose, 5 × 10^6^ MSC equivalents; ~8.6 × 10^8^ particles) or with EVs-free iodixanol vehicle only (control group). EVs were intravenously administered via the tail vein. Animals were evaluated on days 7, 14, or 28 by cytometric, histological and/or quantitative PCR analysis. A single dose of BM-MSCs-derived EVs attenuated bleomycin-induced damage, improving lung morphology, reducing collagen deposition, and restoring lung architecture. After both 7 and 14 days, treatment with BM-MSCs-derived EVs increased the populations of alveolar macrophages and infiltrated monocytes, while reducing the classical proinflammatory monocytes. The same immunomodulatory effect was demonstrated in myeloid cells derived from the BM. In order to investigate the modulatory effect of EVs on the BM-myeloid/monocyte cell lineage phenotype, these cells were preconditioned with BM-MSCs-derived EVs ex vivo. Proteomic analysis of this cell line, performed by liquid chromatography–tandem mass spectrometry (LC-MS/MS), demonstrated that treatment with BM-MSCs-derived EVs exerts its protective effects, at least in part, by reprogramming the phenotypic profile of myeloid cells derived from BM toward a nonclassical phenotype. Subsequently, BM-derived myeloid cells preconditioned with EVs ex vivo were administered to mice with pulmonary fibrosis. After receiving bleomycin, animals received two doses of BM-derived myeloid cells preconditioned with EVs on days 0 and 3. Treatment with this cell population reduced collagen deposition, restored lung architecture, and reduced inflammation. These data demonstrate that the effects of BM-MSCs-derived EVs mediated, at least part, the myeloid cells reprogram toward a proregulatory phenotype, thus reducing infiltration into the lungs of proinflammatory and profibrotic monocytes [150].

#### 6.1.2. Animal Models of BPD

Bronchopulmonary dysplasia (BPD) is a complex chronic lung disease that is common in premature infants, especially in very low birth weight and extremely low birth weight infants with an incidence of 30–40% and 54.1%, respectively [2]. In the early postnatal period of preterm infants, the pulmonary cells in the late canalicular or early saccular stage are in a highly proliferative state, leading to the lack effective alveolar gas exchange [2]. BPD results in a lung injury and abnormal lung repair, characterized by a decrease in the number of alveoli, abnormal morphology, uneven ventilation distribution, and abnormal development of the vascular system of the lung [2]. Similarly to chILD, the consequences of lung immaturity remain through-out childhood and can often lead to chronic respiratory diseases characterized by parenchymal fibrosis and alveolar and vascular growth block [2,151], For this reason, BPD may represent a model to document the efficacy of MSCs treatment in ILD.

Moreira et al. [152] investigated the efficacy and safety of intranasal MSCs infusion in a BPD model. The cells were obtained from the gelatinous tissue of Wharton’s umbilical cord from a healthy human infant. The rat pups at postnatal day 4 were randomly divided into four groups. One group was maintained in normoxic conditions (21% O_2_) for 21 days (controls group). The remaining groups underwent continuous hyperoxia (60% O_2_) for 4 days to induce the BPD model. In the study, one group of BPD rats received nothing (BPD group), one group was treated with vehicle, and one group received MSCs (20 μL containing 5 × 10^5^ cells/kg) on days 4, 12, and 20. Morphometric pulmonary analyses showed that the administration of MSCs restored alveolarization, vascularization, and pulmonary remodeling, which, on the contrary, are altered in hyperoxic conditions. In pulmonary homogenates, treatment with MSCs increased vascular endothelial growth factor mRNA expression compared to the control group, which did not change compared to the BPD group. An analysis of lung tissue genes and proteins suggested that MSCs exert their beneficial effects, at least in part, by modulating genes involved in angiogenesis, immunomodulation, wound healing, and cell survival. Therefore, the data from this study showed that MSCs can improve the outcomes of BPD infants. Notably, these findings demonstrated that the experimental intranasal route of administration is a feasible, noninvasive, and efficacious route that may have clinical applicability [152].

The beneficial effect of MSCs-derived EVs was also demonstrated by Portionato et al. [153] in a mouse model of BPD induced by exposure to hyperoxia in the first 2 weeks postnatally. The newborn rats were randomly divided into four groups: one group of animals was raised for six weeks in normoxic conditions, as a control group. The experimental groups were exposed to 60% hyperoxia for 2 weeks, and for another 4 weeks to normoxic conditions. Half of the animals in the experimental group received saline (sham-treated animals), while the others were treated with MSC-derived EVs (0.64 × 10^10^ particles) or with MSCs (6 × 10^6^ cells/kg) on postnatal days 3, 7, and 10 via intratracheal administration. MSCs-derived EVs delivery increased the number of alveoli, their surface area, and the proliferation index, which were decreased in the hyperoxic conditions. Furthermore, the treatment reduced mean alveolar volume, mean linear intercept, and fibrosis. The medial thickness index for vessels <100 µm reduced in MSCs-derived EVs, which increased in hyperoxic rats compared to the normoxic group. EVs also prevented the reduction in CD163-positive macrophages in both interstitial/alveolar and perivascular populations, which was instead induced in hyperoxic conditions. Intratracheal administration appears to be intriguing for translation in the future, probably due to its capacity to directly target organs for treatment. These data support the use of MSCs-derived EVs as a cell-free approach to improve altered alveolarization and remodeling of the pulmonary artery even after long-term treatment in preterm-born human infants [153]. These findings suggested that most of MSCs’ beneficial effects are mediated by paracrine signaling, pointing to the feasibility of using MSC secretome as a therapeutic tool. However, both the toxicity and biodistribution of MSCs-derived EVs need further investigation in animal models before they can be used as first-in-human patients.

Th paracrine effects of MSCs are mediated by the release of immunomodulatory and growth factors that have been identified in the CM [154]. In this regard, Hansmann et al. [155] evaluated the effects of the CM in BPD management. In order to induce the BPD pattern, newborn FVB mice were exposed to 75% O_2_ for 14 days, while a control group was kept in normoxic conditions. After 14 days of exposure to hyperoxic conditions, the animals were treated with a dose of BM-MSCs-derived CM isolated from the femurs and tibias of FVB mice, at a concentration of 10 μg of BM-MSCs-derived CM protein per mouse. A group of animals subjected to hyperoxic conditions, instead, was treated with mouse lung fibroblasts (MLF)-derived CM as a control group. CM was administered intravenously either through the superficial temporal vein or the jugular vein. Compared to treatment with MLF-derived CM, the administration of BM-MSCs-derived CM improved the hyperoxia-induced pathogenesis of BPD. BM-MSCs-derived CM reduced alveolar damage, septal thickening, and myofibroblast infiltration, and improved lung function. Treatment with BM-MSCs-derived CM attenuated pulmonary hypertension, right ventricular hypertrophy, and peripheral pulmonary artery pressure muscularization related to hyperoxia-induced BPD. Furthermore, pulmonary artery pruning lead by hyperoxia was ameliorated by a single intravenous dose of MSC-derived CM, highlighting the angiogenic and vasculogenic effect of MSC-derived CM. Therefore, the use of CM derived from MSCs could be a valid therapeutic option for BPD and other chronic lung diseases; however, future studies will be needed to understand the mechanisms underlying their beneficial effects [155].

The results of all these studies (Table 4) showed the beneficial effects MSCs transplantation in tissue recovery and lung function following pediatric fibrotic lung disease. These findings also highlight that MSCs’ beneficial effects are mediated, at least in part, by paracrine signaling, demonstrating the feasibility of using MSC secretome as a therapeutic tool. However, further preclinical studies are required to evaluate the long-term safety of systemic transplantation with MSCs and the toxicity and biodistribution of MSCs secretome before it can be used in humans, especially when considered for pediatric use.

### 6.2. Clinical Studies

Recent evidence showed that MSCs represent a valid therapeutic approach for the treatment of pulmonary diseases in adult patients, including chronic obstructive pulmonary disease, silicosis, acute respiratory distress, and idiopathic pulmonary fibrosis [146,156,157,158,159]. To date, in the pediatric population, the therapeutic effects of MSCs on respiratory diseases were evaluated in BPD that may be considered as potential model of interstitial diseases and thus discussed.

#### 6.2.1. Phase 1 Clinical Trials

Chang et al. [160] in a phase I clinical trial (NCT01297205), evaluated the safety, feasibility, and efficacy of the transplant of human umbilical cord blood derived MSCs (hUCB-MSCs) in premature BPD children. In the study, the recruited nine infants of gestational age 24–26 weeks (extremely premature) and at high risk of developing BPD, with damaged respiratory conditions and on ventilatory support. Three infants received a low dose of hUCB-MSCs (1 × 10^7^ cells/kg), and six received high-dose hUCB-MSCs (2 × 10^7^ cells/kg). Cells were intratracheally delivered into the left and right lungs. Cell transplantation was found to be safe: neither serious adverse effects nor dose-limiting toxicity up to 84 days following transplantation were observed. Only three out of nine children developed moderate BPD, highlighting the beneficial effects of cell transplantation. hUCB-MSCs transplantation also reduced the duration of intubation, the mean respiratory index 3 days after transplantation, steroid use, and positive airway pressure. Additionally, compared to baseline or day 3 after cell infusion, at day 7 in tracheal aspirates, treatment reduced levels of IL-1, IL-6, IL-8, IL-10, matrix metalloproteinase (MMP)-9, TGF-β, and TNF-α. Therefore, the data from this study demonstrated that intratracheal hUCB-MSCs delivery may be safe and feasible in preterm infants, and may be effective in reducing BPD severity [160].

A long-term follow-up study is underway (NCT01632475) to evaluate the safety and feasibility of the long-term treatment with these cells of premature infants with BPD enrolled in the previous trial. The primary aim of the study led by Ahn et al. [161] is to evaluate the long-term safety of MSC transplantation in infants up to 24 months of age. Only one in nine children died six months after treatment, but this adverse event was not related to MSC transplantation. None of the remaining infants experienced treatment-related adverse events. Therefore, intratracheal transplantation with hUCB-MSCs would appear to be safe in premature infants at high risk of developing BPD up to 2 years of age. Compared to the comparison group, infants treated with hUCB-MSCs no longer needed supplemental oxygen. After 18–24 months, the infants demonstrated weight gain that could be related to better long-term neurodevelopmental outcomes [161].

#### 6.2.2. Phase 2 Clinical Trials

In order to further evaluate the efficacy and safety of stem cell therapy for BPD, a long-term clinical trial is underway for children up to the age of 5 years (NCT02023788) for patients who completed the earlier part of the previous phase I clinical trial (NCT01632475). Ahn et al. [162] performed a larger, double-blind, randomized, phase II clinical trial aimed at evaluating the therapeutic efficacy of transplantation with hUCB-MSCs (NCT01828957). The study enrolled 60 extremely premature infants, aged between 23 and 28 gestational weeks, with BPD. In the experimental group, infants received hUCB-MSCs in step 6 (1 × 10^7^ cells/kg), administered intratracheally via a gavage tube in two fractions in the left and right lungs. The placebo group only received an equal volume of saline. Treatment with hUCB-MSCs reduced levels of IL-1β, IL-6, IL-8, TNF-α, and MMP-9 at day 7 compared to the control group. However, the treatment did not improve the death outcome or disease progression. Treatment with hUCB-MSCs improved the outcome of severe BPD from 53% to 19% in the 23–24 gestational weeks subgroup of infants. Conversely, the severity of the disease was not improved in the subgroup of patients aged from 25 to 28 gestational weeks. Therefore, these data showed that hUCB-MSCs transplantation is effective and feasible; however, the small number of samples does not allow establishing the efficacy in preterm infants aged from 23 to 28 gestational weeks [162]. Therefore, a further larger phase II study is underway recruiting infants aged from 23 to 24 gestational weeks (NCT03392467). Sixty premature infants within 13 days of postnatal age were recruited in the trial. The experimental group will receive hUCB-MSCs (1 × 10^7^ cells/kg), while one group will receive a saline solution (control group). The results of this study will help to understand the efficacy and safety of hUCB-MSCs for the treatment and prevention of severe BPD in premature infants. The subjects who passed phase II of the trial (NCT01828957) were recruited into the follow-up study, which aims to monitor participants up to the age of 5 years (NCT01897987). The main trial aim is to evaluate the respiratory outcome following transplantation with hUCB-MSCs compared to the control group.

Another open-label dose-escalation trial (phase I/II), led by Powell et al. [163] aimed to investigate the safety of single-dose intratracheal administration of hUCB-MSCs in extremely low birth weight preterm infants with BPD (NCT02381366). The study enrolled 12 premature infants born at 23–27 weeks of gestation with a high risk of BPD. One group of infants received hUCB-MSCs at the lowest dose (1 × 10^7^ cells/kg), and the other group at the highest dose (2 × 10^7^ cells/kg). The administration of hUBC-MSCs, at both doses, was well-tolerated by all patients with no signs of toxicity during the 72 h of observation. No serious adverse events were recorded during the 84 days of the study. Therefore, treatment with hUCB-MSCs proved to be safe and feasible [163].

Based on these promising results, Wu et al. [9] performed a phase II study, with respective control groups, to further investigate the safety and efficacy of the use of allogeneic hUCB-MSCs in children with severe BPD (NCT03601416). The study recruited 72 children up to the age 1 year with moderate or severe BPD undergoing traditional supportive treatments. Participants were treated intravenously with low-dose (2.5 × 10^6^ cells/kg) or high-dose (5 × 10^6^ cells/kg) hUCB-MSCs. The end date of the trial is expected to be December 2021. The study results will help prove the long-term safety and efficacy of hUCB-MSCs. They will also allow the ability of treatment to improve lung structure impairment by exploring its potential therapeutic use in the management of severe childhood BPD [9].

#### 6.2.3. Active Clinical Trials

Currently, 13 additional clinical trials of MSCs therapy for BPD have been registered with ClinicalTrials.gov. Among them, five phase I studies (NCT02443961, NCT03631420, NCT01207869, NCT04255147, and NCT03683953) are active in not-recruiting status. All five registered clinical trials involve the administration of MSC via intratracheal or intravenous routes at doses ranging from 1 to 30 × 10^6^ cells/kg of body weight in children (1–37 weeks) at high risk of BPD. Seven phase I/II studies (NCT03774537, NCT03558334, NCT03873506, NCT04003857, NCT03378063, NCT04062136, and NCT03645525) are in recruiting status. These trials foresee the administration of hUCB-MSCs by an intratracheal or intravenous route at doses ranging from 1 × 10^6^ to 2 × 10^7^ cells/kg of body weight in children (from 3 days to 5 years of age) at high risk of BPD. The study results will better assess the safety, feasibility, and efficacy of MSCs for the prevention and treatment of premature infants at high risk of BPD.

#### 6.2.4. Terminated Clinical Trial

A placebo-controlled trial (NCT03857841) intended to evaluate the safety of intravenous infusion of BM-MSCs-derived EVs (UNEX-42) in preterm neonates (from 3 to 14 days postnatal) at high risk of BPD. The BM-MSCs-derived EVs were administered at doses of 20, 60, or 200 pmol phospholipid/kg body weight. The results of this study will be useful for understanding the safety and tolerability of BM-MSCs-derived EVs up to 10 months after treatment. Furthermore, at the end of this post-treatment phase, patients will be included in the next phase and will be monitored until they reach 1 year of age to also evaluate the long-term effects of pediatric treatment with cell derivatives.

#### 6.2.5. Clinical Trial in Child with Chronic Respiratory Failure

In a brief report, Calcaterra et al. [164] isolated MSCs from the lung tissue of a male infant presumed to have congenital lobar emphysema and filamin A (*FLNA*) gene mutation. MSCs isolated from this child’s lung tissue exhibited the same characteristics as MSCs and the ability to differentiate into osteoblasts. Instead, these MSCs, compared to the control BM-MSCs, showed low migration capacity, which could be linked to FLNA deficiency. Therefore, these data highlight the important role that may have been played by dysfunctional lung MSCs in impaired lung development and in the formation of emphysematous lesions. In addition, they may also have been responsible for the altered matrix remodeling that induced progressive fibrotic lung disease [164]. Based on this evidence, in a clinical study performed by Pelizzo et al. [11], the effects of repeated intravenous administration of allogeneic BM-MSCs were evaluated in a child with progressive obstructive pulmonary disease associated with an *FLNA* gene mutation [11]. The mutation in this gene was recently associated with lung growth abnormalities, which, in several cases, progress to interstitial lung disease [165]. In this work, a 32-day old male child was hospitalized with respiratory distress and suspected congenital lung malformation. He underwent lobar resection of the damaged lung segments, noninvasive mechanical ventilation at 11 months, followed by a tracheostomy after 1 month. Therefore, at the age of 18 months, due to severe and irreversible chronic respiratory failure, the child underwent salvage therapy with allogeneic BM-MSCs. The allogeneic BM-MSCs were isolated and expanded ex vivo from healthy donor bone marrow. The child received four infusions of MSCs (1 × 10^6^ cells/kg) intravenously 4 weeks apart. Before each infusion and 1 month after the last one, peripheral blood and mononuclear cells were collected. Treatment with allogeneic MSCs greatly improved the child’s respiratory condition. Furthermore, the positive effects observed after the second dose suggested the need for serial administrations rather than single injections. The mechanisms used by MSCs to exert these beneficial effects are, at least in part, mediated by paracrine immunomodulatory capacity on the recipient lung tissue. After the second MSCs infusion, decreased levels of Th17 and the consequent normalization of the T_reg_/Th17 balance were observed, which is usually dysregulated in various lung diseases. The increase in PHA-induced PBMC proliferation after injections of MSCs and the increase in the percentages of B lymphocytes support the involvement of MSCs in immune-mediated processes. The data from this work also showed that intravenous administration was well-tolerated: no serious adverse events were reported in the child. Therefore, the intravenous route of administration, taking advantage of the pulmonary first-pass effect that characterizes MSCs, could represent the optimal route of administration in lung diseases’ treatment. In conclusion, this clinical study supports the use of serial infusions of MSCs in the pediatric treatment of mutated *FLNA*-associated respiratory failure [11].

In all studies, treatment with MSCs was authorized after receiving informed parental consent of enrolled children. The clinical trials (phases I/II) described above (Table 5) aim to show the safety and/or efficacy of MSC use as a therapeutic tool in pediatric BPD and chronic respiratory failure.

## 7. Future Perspectives

Repetitive micro injury to the AEC, which leads to aberrant repair during tissue regeneration, is a central paradigm of chILD [95]. Currently medical treatment of chILD is empirical with varying efficacy. Prospective therapies must be developed to ameliorate the prognosis of chILD. MSCs may be considered a promising cellular source to prevent disease progression or to revert established lung fibrosis, suppressing inflammation and supporting alveolar repair. The role of MSCs in the reparative and regenerative processes can also be considered. The plasticity of fibrosis [102] in the pediatric context of pulmonary disorders associated with fibrosis might represent an additional opportunity for cell therapy. A multidisciplinary team approach represents the standard of care for chILD diagnosis and management; it should become a core standard in post-operative treatment of cystic lesions and pneumothorax relapses in chronic pediatric lung disease. Combined treatment including medicaments, cell therapy, and surgery could offer a novel therapeutic approach in children, even in case of severe lung hypoplasia related to complex congenital malformations such as pulmonary malformations, congenital diaphragmatic hernia, or lung volume resection in *FLNA* gene mutations. The poor prognosis when prenatally diagnosed could be mitigated from birth by the use of combined therapy including surgery and stem cells with the aim of promoting lung growth.

## 8. Conclusions

Cellular therapy represents a potential treatment for chILD. Even though beneficial effects have been reported in clinical and preclinical studies, the optimal dosage, cell type, cell source, and route of administration have not yet been fully elucidated. Due to their tissue-regenerative and immunomodulatory properties, MSCs combined with other therapy or alone could be considered an innovative approach for the repair and regeneration of the lung during disease progression and/or as post-surgical lung support after pulmonary resection due to severe respiratory complications in chronic lung diseases.

## Figures and Tables

**Table 1:** Classification of children’s interstitial lung disease according to Rice et al. [97].

Disorders More Prevalent in Infants	Disorders More Prevalent in Children
Diffuse developmental disorders (acinar dysplasia, alveolar capillary dysplasia, congenital alveolar dysplasia)	Disorders related to systemic disease (Langerhans cell histiocytosis, related to acquired heart disease, storage disease/endogenous lipid pneumonia)
Growth abnormalities (alveolar hypoplasia, chronic neonatal lung disease, related to chromosomal disorders, related to congenital heart disease)	Disorders of the normal host (eosinophilic bronchiolitis/pneumoniae, infection/post infectious processes, hypersensitivity pneumonitis, aspiration pneumonia)
Specific conditions of undefined etiology (neuroendocrine cell hyperplasia of infancy, pulmonary interstitial glycogenosis)	Disorders of the immunocompromised host: (opportunistic infection, transplant-related)
Surfactant protein disorders	Disorders masquerading as ILD (pulmonary hypertension, veno-occlusive disease, lymphatic disorders capillary hemangiomatosis, thromboembolic disease, vasculitis)
	Lymphoproliferative disease (lymphoid interstitial pneumonia, diffuse lymphoid hyperplasia, lymphomatoid granulomatosis)
	Small airways disease (chronic bronchiolitis, obliterative bronchiolitis, follicular bronchiolitis)
	Interstitial pneumonias unrelated to surfactant protein disorder (organizing pneumonia, diffuse alveolar damage, usual interstitial pneumonia)
	Other patterns of diffuse lung disease (hemosiderosis, alveolar microlithiasis, sarcoidosis)

**Table 2:** Genetic mutations associated with children’s interstitial lung disease.

Genetic Mutation	Inheritance	Lung Involvement
SFTPB (surfactant protein B deficiency)	Autosomal recessive	Surfactant disorder
SFTPC (surfactant protein C mutation)	Autosomal dominant	Surfactant disorder
CSF2RB (colony stimulating factor 2 receptor β)	Autosomal recessive	Pulmonary alveolar proteinosis
CSF2RA (colony stimulating factor 2 receptor α)	X-linked	Pulmonary alveolar proteinosis
ABCA3 (ATP-binding cassette-family A-member 3)	Autosomal recessive	Deficit surfactant
COPA (coatomer associated protein subunit alpha)	Autosomal dominant	General disorder including lung
FLNA (Filamin A)	X-linked recessive	General disorder including lung
FOXF1 (forkhead box F1)	Autosomal dominant	Alveolar capillary dysplasia
GATA2 (GATA Binding Protein 2)	Autosomal dominant	Pulmonary alveolar proteinosis
MARS (metionil-transfer RNA sintetasi)	Autosomal recessive	Pulmonary alveolar proteinosis
NKX2-1 (NK2 homeobox 1)	Autosomal dominant	Interstitial lung disease
NSMCE3 (non-structural maintenance of chromosome element 3 homolog)	Autosomal recessive	Immunodeficiency
OAS1 (oligoadenylate synthetase 1)	Autosomal dominant	Pulmonary alveolar proteinosis
SLC7A7 (solute carrier family 7 member 7)	Autosomal recessive	Surfactant disorder
TBX4 (T-box transcription factor 4)	Autosomal dominant	Acinar dysplasia
TMEM173 (transmembrane protein 173)	Autosomal dominant	Lung fibrosis with general inflammation

**Table 3:** Severity-of-illness score according to Fan et al. [106].

1. Asymptomatic
2. Symptomatic, normal room air oxygen saturation under all conditions
3. Symptomatic, normal resting room air saturation but abnormal saturation (90%) with sleep or exercise
4. Symptomatic, abnormal resting room air saturation (90%)
5. Symptomatic with pulmonary hypertension

**Table 4:** Synthesis of the studies that evaluate the role of MSCs in several animal models of pediatric pulmonary fibrosis (PF).

In Vivo Models	Cell Therapy	Dose	Route of Administration	Intervention	Resuts	Ref.
Newborn Sprague-Dawley rats Hyperoxia-induced BPD	Human umbilical cord Wharton’s Jelly-derived MSCs transplantation	5 × 10^5^ cells/kg	Intranasal delivery	Multiple administration on days 4, 10, and 20, after 3 weeks from induction	Improvement of alveolarization, vascularization, and pulmonary remodeling Increased of the mRNA expression of vascular endothelial growth factors Modulation of genes involved in angiogenesis, immunomodulation	[152]
Severe combined immunodeficiency–beige mice Hyperoxia-induced BPD	hUC-MSCs transplantation	0.1, 0.5, or 1 × 10^6^ cells/kg	Intranasal or intraperitoneal administration	Single dose on postnatal day 5, during induction	Recovery of lung compliance, pressure-volume loop and elastance Enhancement of the thickness of the alveolar septum	[149]
Wild-type Sprague-Dawley rats Hyperoxia-induced BPD	MSCs-derived EVs or MSCs administration	0.64 × 10^10^ particles	Intratracheal delivery	Multiple administration on days 3, 7, and 10 after induction	Enhancement of the number of alveoli, alveolar surface area, and proliferation index Decrease of the mean alveolar volume, mean linear intercept, and fibrosis was decreased Reduction the medial thickness index for vessels	[153]
		6 × 10^6^ cells/kg				
Mouse pups FVB Hyperoxia-induced BPD	BM-MSCs-derived CM or MLF-derived CM	CM containing 10 μg protein	Intravenous administration	A single dose after 14 days of induction	Reduction of the alveolar damage, septal thickening, myofibroblast infiltration Improvement of the lung function Reduction of the pulmonary hypertension, right ventricular hypertrophy, and peripheral pulmonary artery pressure muscularization	[155]

AD-MSCs—adipose-derived mesenchymal stem cells; TNF-α—tumor necrosis factor-alpha; IL—interleukin; Bax—B-cell lymphoma protein 2-associated X; Bcl-2—B-cell lymphoma protein 2; LSCs—lung spheroid cells; BPD—bronchopulmonary dysplasia; MSCs—mesenchymal stromal cells; hUC-MSCs—human umbilical cord-derived MSCs; BM-MSCs—bone-marrow-derived mesenchymal stem cells; EVs—extracellular vesicles; CM—conditioned medium; MLF—mouse lung fibroblasts; PF—pulmonary fibrosi.

**Table 5:** Synthesis of the clinical trials of stem cell therapy in pediatric pulmonary diseases (https://clinicaltrials.gov/, accessed on 10 November 2021). The table shows the efficacy and safety of stem cell therapy in the management of BPD and mutated FLNA-associated respiratory failure.

Identifier	Phase	Subjects	Cells Therapy	Route of Administration	Intervention/ Treatment	Efficacy	Security	Ref.
NCT01297205	Phase 1 (completed)	9 premature infants (up to 14 days) at high risk for BPD	hUCB-MSCs transplantation (PNEUMOSTEM)	Intratracheal delivery	1 × 10^7^ or 2 × 10^7^ cells/kg	Improvement of the respiratory condition Reduction of IL-1, IL-6, IL-8, IL-10, MMP-9, TGF-β, and TNF-α levels, in tracheal aspirates	Well-tolerated and no serious adverse events	[160]
NCT01632475	Phase 1 (active, not recruiting)	9 premature infants (up to 14 days) at high risk for BPD	hUCB-MSCs transplantation (PNEUMOSTEM)	Intratracheal delivery	1 × 10^7^ or 2 × 10^7^ cells/kg	Improvement of the respiratory condition Weight gain	No adverse events	[161]
NCT02023788	Phase 1 (completed)	8 premature infants (from 45 to 63 months) at high risk for BPD	hUCB-MSCs transplantation (PNEUMOSTEM)	Intratracheal delivery	1 × 10^7^ or 2 × 10^7^ cells/kg	-	-	-
NCT01828957	Phase 2 (completed)	69 premature infants (up to 14 days) at high risk for BPD	hUCB-MSCs transplantation (PNEUMOSTEM)	Intratracheal delivery	1 × 10^7^ cells/kg	Decrease of the IL-1β, IL-6, IL-8, TNF-α, and MMP-9 levels. Improvement of the outcome of severe BPD	No adverse events	[162]
NCT03392467	Phase 2 (recruiting)	60 premature infants (up to 13 days) with severe BPD	hUCB-MSCs transplantation (PNEUMOSTEM)	Intratracheal delivery	1 × 10^7^ cells/kg	-	-	-
NCT01897987	Phase 2 (completed)	62 premature infants at high risk for BPD	hUCB-MSCs transplantation (PNEUMOSTEM)	Intratracheal delivery	1 × 10^7^ cells/kg	-	-	-
NCT02381366	Phase 1/2 (completed)	9 premature infants (up to 14 days) at high risk for BPD	hUCB-MSCs transplantation (PNEUMOSTEM)	Intratracheal delivery	1 × 10^7^ or 2 × 10^7^ cells/kg	-	Well-tolerated without signs of toxicity No serious adverse events	[163]
NCT03601416	Phase 1 (not yet recruiting)	72 children (up to 1 year) with moderate and severe BPD	Allogenic hUC-MSCs transplantation	Intravenous administration	2.5 × 10^6^ or 5 × 10^6^ cells/kg	-	-	[9]
NCT02443961	Phase 1 (active, not recruiting)	10 preterm newborns (from 1 month to 28 weeks) at high risk of BPD	MSCs transplantation	-	3 doses of 5 × 10^6^ cells/kg	-	-	-
NCT03631420	Phase 1 (active, not recruiting)	9 infants (up to 51 days) at high risk for BPD	hUC-MSCs transplantation	-	3 × 10^6^, or 10 × 10^6^, or 30 × 10^6^ cells/kg	-	-	-
NCT01207869	Phase 1 (active, not recruiting)	9 extremely premature infants (up to 6 months) with severe BPD	hUC-MSCs transplantation	Intratracheal delivery	3 × 10^6^ cells/kg	-	-	-
NCT04255147	Phase 1 (not yet recruiting)	9 extremely premature infants (up to 21 days) at risk of BPD	Allogeneic UC-MSCs transplantation	Intravenous administration	1 × 10^6^, or 3 × 10^6^, or 10 × 10^6^ cells/kg	-	-	-
NCT03683953	Phase 1 (not yet recruiting)	200 infants (28–37 weeks) with BPD	MSCs transplantation	Intratracheal delivery	25 × 10^6^ cells/kg, administrated on 14 days after birth	-	-	-
NCT03774537	Phase 1/2 (recruiting)	20 preterm infants (up to 14 days) at high risk for BPD	hUC-MSCs transplantation	Intravenous administration	1 × 10^6^ or 5 × 10^6^ cells/kg	-	-	-
NCT03558334	Phase 1/2 (recruiting)	12 premature infants with moderate and severe BPD	hUC-MSCs transplantation	Intravenous administration	1 × 10^6^ or 5 × 10^6^ cells/kg	-	-	-
NCT03873506	Phase 1 (recruiting)	30 premature infants (from 1 month to 5 years) with moderate and severe BPD	hUC-MSCs transplantation	Intravenous administration	1 × 10^6^ or 5 × 10^6^ cells/kg	-	-	-
NCT04003857	Phase 1 (recruiting)	60 premature infants (6–60 months) with BPD	hUC-MSCs transplantation	Intratracheal delivery	1 × 10^7^ cells/kg	-	-	-
NCT03378063	Early Phase 1 (recruiting)	100 preterm infants (1–3 months) with BPD	hUCB-MSCs transplantation	-	-	-	-	-
NCT04062136	Phase 1 (recruiting)	10 infants (1–6 months) with BPD	hUC-MSCs transplantation	Intravenous administration	Two injections at a dose of 1 × 10^6^ administered one week apart	-	-	-
NCT03645525	Phase 1/2 (Recruiting)	180 extremely preterm infants at high risk for BPD	hUC-MSCs transplantation	Intratracheal delivery	2 × 10^7^ cells/kg	-	-	-
NCT03857841	Phase 1 (Terminated)	3 preterm neonates (3–14 days) at high risk for BPD	BM-MSCs-derived EVs (UNEX-42) administration	Intravenous administration	20, 60, or 200 pmol phospholipid/kg	-	-	-
-	-	1 infants (32-day-old) with mutated *FLNA*-associated respiratory failure	Allogeneic BM-MSCs transplantation	Intravenous administration	4 infusions of MSCs at 1 × 10^6^ cells/kg dose, 4 weeks apart	Improvement of the respiratory condition. Reduction of the Th17 levels and normalization of the Treg/Th17 balance	Well-tolerated No serious adverse events	[11]

BPD—bronchopulmonary dysplasia; MSCs—mesenchymal stromal cells; hUCB-MSCs—human umbilical cord blood-derived MSCs; IL—interleukin; MMP—matrix metalloproteinase; TGF-β1—transforming growth factor-beta 1; TNF-α—tumor necrosis factor-alpha; hUC-MSCs—human umbilical cord-derived MSCs; hUCB-MSCs—human umbilical cord blood-derived MSCs; EVs—extracellular vesicles; FLNA—filamin A.

## Data Availability

Not applicable.
